# Coherent Functional Modules Improve Transcription Factor Target Identification, Cooperativity Prediction, and Disease Association

**DOI:** 10.1371/journal.pgen.1004122

**Published:** 2014-02-06

**Authors:** Konrad J. Karczewski, Michael Snyder, Russ B. Altman, Nicholas P. Tatonetti

**Affiliations:** 1 Biomedical Informatics Training Program, Stanford University School of Medicine, Stanford, California, United States of America; 2 Department of Genetics, Stanford University School of Medicine, Stanford, California, United States of America; 3 Department of Bioengineering, Stanford University School of Medicine, Stanford, California, United States of America; 4 Department of Biomedical Informatics, Department of Systems Biology, and Department of Medicine, Columbia University, New York, New York, United States of America; Georgia Institute of Technology, United States of America

## Abstract

Transcription factors (TFs) are fundamental controllers of cellular regulation that function in a complex and combinatorial manner. Accurate identification of a transcription factor's targets is essential to understanding the role that factors play in disease biology. However, due to a high false positive rate, identifying coherent functional target sets is difficult. We have created an improved mapping of targets by integrating ChIP-Seq data with 423 functional modules derived from 9,395 human expression experiments. We identified 5,002 TF-module relationships, significantly improved TF target prediction, and found 30 high-confidence TF-TF associations, of which 14 are known. Importantly, we also connected TFs to diseases through these functional modules and identified 3,859 significant TF-disease relationships. As an example, we found a link between MEF2A and Crohn's disease, which we validated in an independent expression dataset. These results show the power of combining expression data and ChIP-Seq data to remove noise and better extract the associations between TFs, functional modules, and disease.

## Author Summary

Transcription factors (TFs) are crucial to the precise regulation of many cellular processes and thus, are responsible for many human phenotypes and diseases. Now that the ENCODE project has mapped hundreds of TFs to their genomic binding locations, extracting functional biological signals is the next step in understanding their role in disease. In this paper, we present a novel approach to identifying TF targets and use these targets to find regulatory relationships between TFs and diseases. We present a large open dataset of putative TF-TF interactions and TF-disease associations which includes known connections as well as novel ones. We validate the association of one of our novel TF-disease associations, MEF2A and Crohn's disease, suggesting that our approach generates testable disease association hypotheses. Integrating these datasets will be crucial for understanding phenotypes and complex diseases.

## Introduction

Transcriptional networks are fundamental to many aspects of biology and disease. Gene expression is a carefully controlled process orchestrated by the activities of transcription factors (TFs) which regulate the transcription of each gene. TFs usually do not work in isolation, but instead multiple factors combine in different ways to regulate groups of genes in a concerted, often cooperative fashion [1]–[6]. The ENCODE project has begun to determine the binding locations of many transcription factors using chromatin immunoprecipitation (ChIP) followed by high-throughput sequencing (ChIP-Seq) [7], [8].

Despite the abundance of data about the genomic binding sites for transcription factors, determining transcription factor targets and when factors are active remains challenging. ChIP-Seq measurements can be noisy and reflect the particular condition in which the experiments are performed. Collecting more data alone will not solve this problem. As additional experiments are performed for each factor, critical and frequently used binding regions do become apparent, but it is often difficult to determine a signal threshold to distinguish common sites from condition-specific sites and general non-thematic associations from interesting biology. For example, NFκB binds to over 15,000 regions of the genome covering all possible regulatory targets of the factor. But in any given biological context, such as a local cooperative interaction with another transcription factor such as Stat1, only a handful of these genes are actively regulated by NFκB at any one time [4]. This property of TF function gives the illusion that TFs are operating broadly when in fact they perform specific context-dependent functions–in many cases with specific partners. These difficulties conspire to make the regulome challenging to study at a global level.

Thus, to understand transcription factor function, there is a need for computationally-efficient methods to (i) improve TF-target identification, (ii) identify small functional modules that represent context-specific biology, regulated by transcription factors, and (iii) annotate those modules with their functional implications (e.g. the role of the module in human disease).

Recently, a number of methods were developed to derive a network structure to connect sets of genes (modules) to the factors that control their expression [9]–[11]. These methods use gene expression data to derive the most parsimonious regulatory structure. However, because of their computational complexity, they can only account for a limited number of factors, require a specific type of input datasets, and, in their current form, cannot integrate other experimental data (e.g. ChIP-Seq). Thus, these methods may not be sufficient to capture the scale and complexity of the human regulome. Additionally, their usefulness is hampered by an assumption that the activity of the TF can be estimated by its expression, which, the authors of these methods acknowledge is not true in many cases [9]. Efficient approaches with the capability to integrate multiple data modalities are needed in order to properly leverage high-throughput experiments in the study of disease.

**Figure 1:**
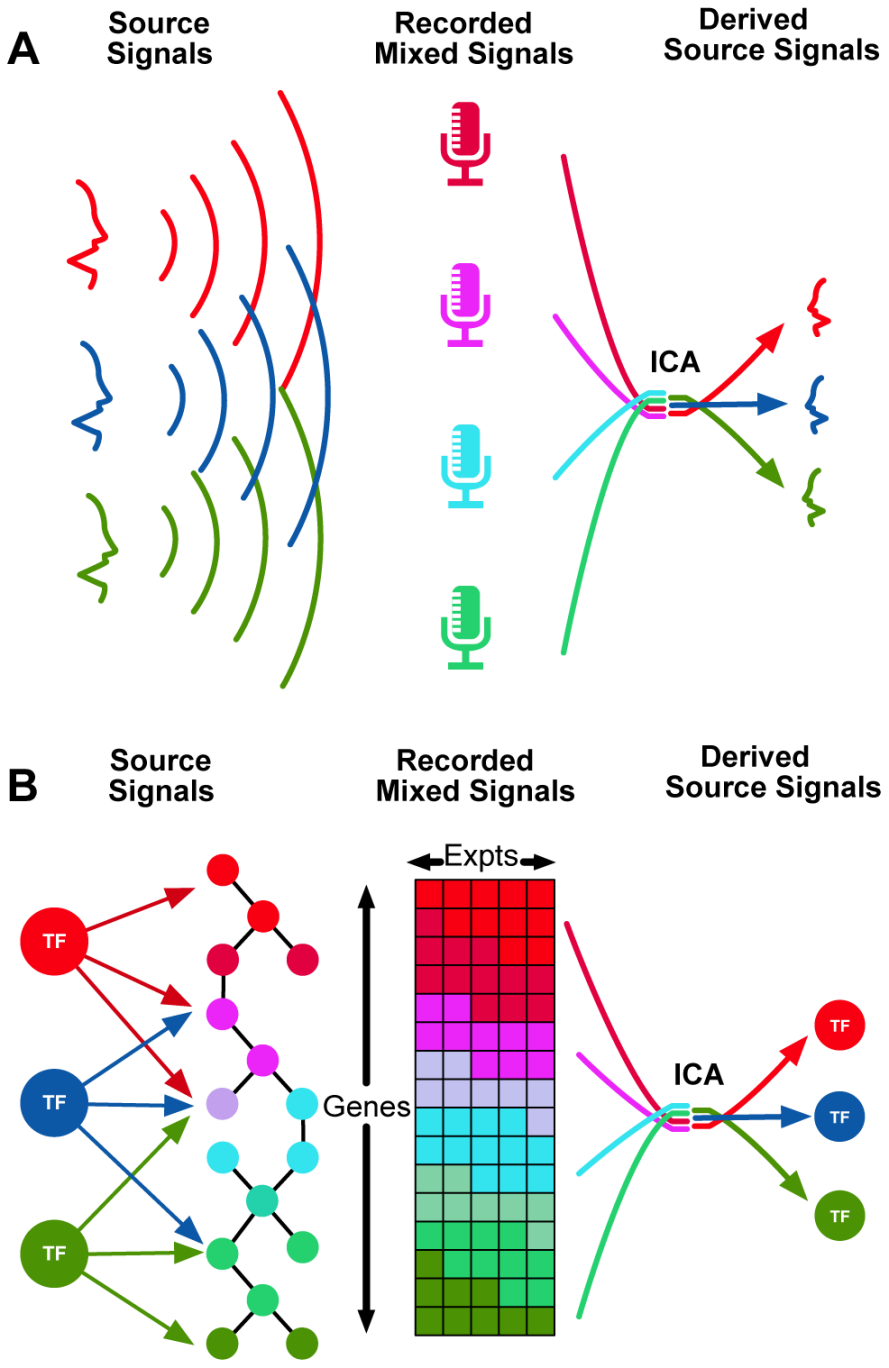
Independent Component Analysis (ICA) can be used to identify transcriptional modules from gene expression data. (A): The classical example of ICA is the “cocktail party problem,” where a number of microphones are placed in a room, capturing a mixture of conversations. Source separation methods such as ICA attempt to deconvolve the recorded mixed signals into their separate source signals (individual conversations). (B): An analogous application involves identifying source signals of transcriptional regulators from complex gene expression measurements.

In this paper, we use factor analysis as a computationally-efficient method to (i) improve the identification of transcription factor targets, (ii) identify functional modules from gene expression data, and (iii) use these modules to annotate transcription factors and connect them to diseases. There are several methods for decomposing expression data to find groups of genes that work together. Network Component Analysis (NCA) is a method for inferring transcription factor activity from expression data [12] and has been used to build regulatory networks for model organisms [13]. However, NCA requires *a priori* knowledge of the regulatory structure which is often not available, and introduces bias in the associations between TFs and functional components. On the other hand, independent component analysis (ICA) is an unbiased and efficient method for deconvolving the signal from a fixed set of sources measured by a set of sensors (Figure 1). In essence, ICA is a computational method for extracting a set of signals from noisy data. When applied to gene expression data – like those recorded by microarrays – ICA can identify coherent functional modules (we refer to each ICA component as a module). Importantly, ICA allows genes to participate in multiple modules and thus has some ability to capture different biological contexts. A set of 423 data-driven modules derived from an ICA of 9,395 human expression microarrays covering a wide diversity of human biology was recently reported [14].

Here, we hypothesize that regulation of each of these ICA-derived modules is controlled by a small set of TFs. Using our method (which we call TFICA), we associated transcription factors to modules and then analyzed the genes contained within each module. Intersecting these target modules with ChIP-Seq binding sites improves target identification and elucidates the functional roles of the factors–both individually and in combination. We compare our approach to traditional methods in three areas: the identification of (i) transcription factor targets, (ii) TF-TF cooperativity, (iii) and the functional roles in the context of various diseases. In each of these cases, we found that our approach significantly outperforms the traditional methods. Further, we found improved performance when our approach is used in combination with traditional methods, implying that we are capturing an independent modality of transcription factor activity. Our data-driven approach is unbiased and computationally efficient enabling systematic identification of novel TF-disease relationships. Finally, we validate one such association between MEF2A and Crohn's disease.

## Results

### Functional modules improve the identification of transcription factor targets

**Figure 2:**
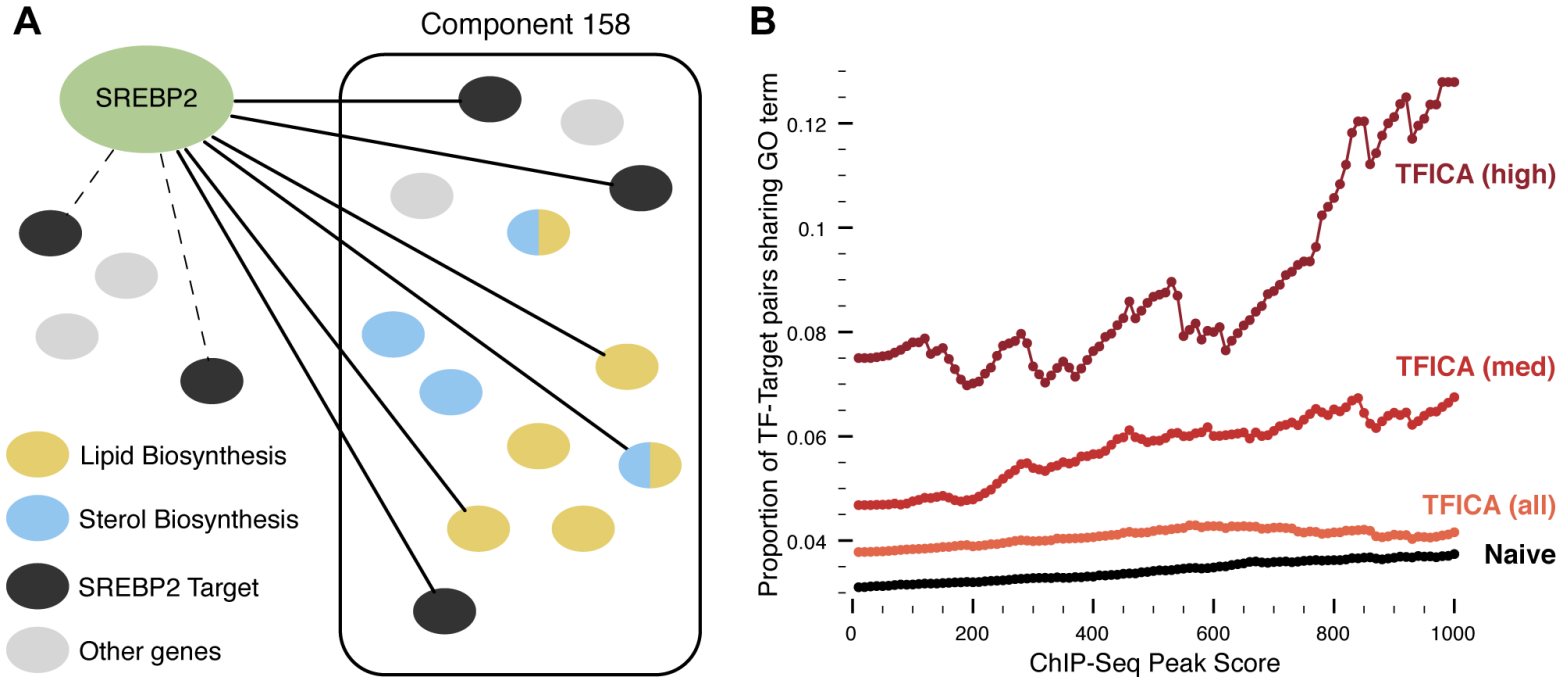
Association of TFs to expression modules. (A): A TF is associated to a module if its targets are significantly enriched in a particular module. TF are connected to their targets using ChIP-Seq data, which may (solid) or may not (dashed) be contained with an expression module. GO annotations (colored blue/yellow) are used in enrichment analysis to associate modules and their factors to functional pathways. (B): We evaluated the quality of TFICA derived TF targets based on the hypothesis that if a TF does regulate a target, then it is more likely that the TF and the target will share a functional annotation. Across ChIP-Seq scores, TFICA outperforms the naive method, and this performance is further increased when only considering high and medium-confidence modules (see text).

We used a set of functional modules derived using ICA [14]. We then used ENCODE ChIP-Seq experimental data to connect transcription factors to individual modules if the factor bound a significant number of genes in that module (Figure 2A; see *Materials and Methods*). For 143 transcription factors and 379 modules, we identified 5,002 significant TF-module associations (with adjusted p<0.01, Fisher's exact test; Figure S1 and Table S1) for an overall FDR of 16.6%. We hypothesized that the components from ICA represent a single regulatory signal analogous to a single voice recorded by set of microphones (as in Figure 1). Thus, modules that associate with only one or a few factors correspond to cleaner signals than those associated with many factors. We identify 31 associations which we called “high-confidence” as there was only one TF significantly associated with the the module and another 142 “medium-confidence” associations, where the module was associated with three or fewer TFs.

We found that many of the modules nearly or fully overlap with targets of only one or a few transcription factors (Figure S3A–C). We found that for 171 modules the top associated TF could account for 80% of the targets in the module (Figure S3D). Additionally, the modules that explain the most variance across the compendium of 9,395 gene expression experiments are significantly associated with a larger number of TFs (Figure S4), and may represent large transcriptional programs.

### Validation of target identification using shared functional annotations

Used in combination with the ChIP-Seq data, we hypothesized that these modules can improve identification of transcription factor targets. Specifically, we believe that putative targets (as determined by ChIP-Seq) which are also contained within significantly associated modules will be more likely to be “true” targets of the TF. To test this hypothesis, we used a set of specific GO terms [15] and we considered shared functional annotation as a proxy for a high quality TF-target association. This strategy has been used successfully in computational evaluation previously [15]. As expected, we found that TF-target pairs, particularly ones with high ChIP-Seq scores, were enriched for pairs with shared functional annotations (p<0.001; Figure 2B). When considering only the targets in the 5,002 modules associated with TFs, we find a significantly higher enrichment for shared annotations (Figure 2B). This enrichment is maintained across all ChIP-Seq peak scores (p<0.05). Additionally, if we only consider targets in the 142 medium-confidence or the 31 high-confidence modules, this enrichment is increased further at all peak score thresholds (p<0.001).

### Validation of target identification using expression correlation

In an analogous fashion to the shared functional annotation approach, we used expression analysis to validate our TF-target associations under the assumption that factor expression can be used as a proxy for factor activity. We hypothesized that for high-confidence modules (i.e. those associated with just one TF) the genes within this module should be controlled predominantly by that single factor. To test this hypothesis we examined the correlation between the expression of the module (see Methods) and the expression of the factor across the compendium of 9,395 gene expression experiments. For example, AP-2γ is the sole significant association for module 360 (OR = 1.66; adjusted p = 0.006) and we found a significant correlation between the expression correlation between module 360 and AP-2γ (Spearman ρ = 0.38, p<0.001).

We systematically evaluated all 5,002 TF-module pairs in this manner (Table S2). We compared our method to two “naive” approaches for generating TF modules: (i) a “best-module” constructed from only the best ChIP-Seq hits for each TF according to their peak scores and (ii) a “matched-module” constructed from a random sample of the TF's ChIP-Seq targets with the same distribution of peak scores as was found in the ICA module. For high-confidence and medium-confidence modules TFICA outperforms the best-module method 60% of the time (binomial p = 0.011), and the matched-module method 77% of the time (binomial p = 3.2e-11; Figure S5). In fact, TFICA outperforms both naive approaches even for those modules associated with many transcription factors (>3). This holds until modules are associated with 40 or more transcription factors, at which point the individual factor expression signal can no longer be observed (Figure S5) and best-module begins to outperform TFICA. TFICA outperforms matched-modules regardless of the number of factor associated to the module (Figure S5). Additionally, six of the modules that are most enriched for a TF's targets are also the most correlated module for that TF (OR = 25.4, Fisher's exact P = 2.8e-7), and 15 are in the top 5% of all modules (OR = 5.6, Fisher's exact P = 1.9e-10). Finally, we found that the top co-expressed module is significantly enriched (Materials and Methods) for ChIP-Seq binding sites for 37 TFs (OR = 4.9, Fisher's exact P = 2.2e-12). We exhaustively evaluated the expression correlation between all TFs and all modules to estimate a null distribution, and found that our TFICA TF-module pairs were significantly more correlated than expected by chance (ρ = 0.05 vs. −0.06; t-test p = 1e-204). Additionally, in all, 327 TF-module pairs remained significantly correlated when compared to an empirically derived TF-specific null distribution (p<0.05; Figure S5).

### TFICA modules are enriched for known transcription factor functional annotations

Many modules connected to TFs were significantly enriched for functional annotations known to be associated with the factor (Table 1 and Figure S2). For instance, sterol regulatory element-binding protein 2 (SREBP2) and module 158 was our most significant TF-module association (Figure 2A; OR = 45.2; 95% CI = (27.8, 71.6); adjusted p = 1e-31). SREBP2 is essential for cholesterol and fatty-acid biosynthesis, and module 158 is significantly enriched for GO terms related to lipid, sterol, cholesterol, and steroid synthesis (adjusted p<0.05; Table 1). In addition, SREBP2 shares many of the same target modules as SREBP1 (Figure S2), which are known regulatory partners.

**Table 1:** Functional modules recapitulate known transcription factor biology.

Rank	Transcription Factor (name)	Functional Module ID	Number of genes in module bound by TF	Number of genes in module	Odds Ratio	Adjusted P Value
1	SREBP2 (Sterol regulatory element-binding protein)	158	30	119	45.2	1E-31
	Module Enriched GO Terms (P<0.05)					
	*lipid biosynthetic process, sterol biosynthetic process, sterol metabolic process, cholesterol metabolic process, steroid biosynthetic process, cholesterol biosynthetic process, steroid metabolic process, isoprenoid biosynthetic process, isoprenoid metabolic process, oxidation reduction, endoplasmic reticulum, endoplasmic reticulum membrane, nuclear envelope-endoplasmic reticulum network, endoplasmic reticulum part, organelle membrane, endomembrane system, microbody, peroxisome*					
	Module Enriched KEGG Pathways (P<0.05)					
	*Steroid biosynthesis, Terpenoid backbone biosynthesis*					
2	GCN5 (Histone acetyltransferase)	104	8	69	33	5.13E-08
	Module Enriched GO Terms (P<0.05)					
	*nucleosome assembly, chromatin assembly, protein-DNA complex assembly, nucleosome organization, DNA packaging, chromatin assembly or disassembly, cellular macromolecular complex assembly, cellular macromolecular complex subunit organization, chromatin organization, chromosome organization, macromolecular complex assembly, macromolecular complex subunit organization, nucleosome, protein-DNA complex, chromatin, chromosomal part, chromosome, intracellular non-membrane-bounded organelle, non-membrane-bounded organelle, DNA binding*					
	Module Enriched KEGG Pathways (P<0.05)					
	*Systemic lupus erythematosus*					
3	GCN5 (Histone acetyltransferase)	62	13	183	20.5	2E-10
	Module Enriched GO Terms (P<0.05)					
	*translational elongation, translation, cytosolic ribosome, ribosome, ribosomal subunit, cytosolic part, ribonucleoprotein complex, cytosol, cytosolic small ribosomal subunit, cytosolic large ribosomal subunit, large ribosomal subunit, small ribosomal subunit, intracellular non-membrane-bounded organelle, non-membrane-bounded organelle, structural constituent of ribosome, structural molecule activity, RNA binding*					
	Module Enriched KEGG Pathways (P<0.05)					
	*Ribosome*					
4	NELFe (Negative elongation factor E)	104	19	69	19.5	2.85E-14
	See annotations for #2					
5	ZNF274 (zinc finger protein 274)	111	71	196	18.6	7.2E-50
	Module Enriched GO Terms (P<0.05)					
	*transcription, regulation of transcription, DNA-dependent, regulation of transcription, regulation of RNA metabolic process, zinc ion binding, DNA binding, transition metal ion binding, metal ion binding, cation binding, ion binding*					
	Module Enriched Interpro Terms (P<0.05)					
	*Zinc finger, C2H2-type/integrase, DNA-binding, Krueppel-associated box, Zinc finger, C2H2-type, Zinc finger, C2H2-like*					
71	NFKB	8	217	257	4.6	1.8E-21
	Module Enriched GO Terms (P<0.05)					
	*cell activation, leukocyte activation, lymphocyte activation, T cell activation, leukocyte differentiation, hemopoietic or lymphoid organ development, hemopoiesis, immune system development, lymphocyte differentiation, positive regulation of lymphocyte activation, positive regulation of T cell activation, T cell differentiation*					
Various	Module significantly associated with 121 Factors	57	Various	159	Various	Various
	Module Enriched GO Terms (P<0.05)					
	*regulation of RNA metabolic process, regulation of transcription, regulation of transcription from RNA polymerase II promoter, regulation of transcription, DNA-dependent, transcription, DNA binding, sequence-specific DNA binding, transcription factor activity, transcription regulator activity*					

Top 5 TF-module associations, as well as NFκB and a general transcription module (associated with 141 different TFs) are shown.

Another example is the association between the transcription factor ZNF274 and module 111. Module 111 includes many zinc finger proteins of which ZNF274 is a known regulator [16]. In addition, ZNF274 clusters near SETDB1 and KAP1 (Figure S2A) and has been shown to recruit both of these transcription factors to repress the expression of other zinc finger proteins [17], [18] (Table 1).

Module 57 was associated with the greatest number of transcription factors (121 different factors; Table 1, Figure S2). This module also contains the greatest number of transcription factors as targets (14 factors). We found this module to be significantly enriched for DNA binding, regulation of transcription, and transcription regulator activity (among other regulatory terms; Table 1), and may represent a master regulatory module of other TFs.

### Transcription factors that share associated modules show evidence of potential interactions

**Figure 3:**
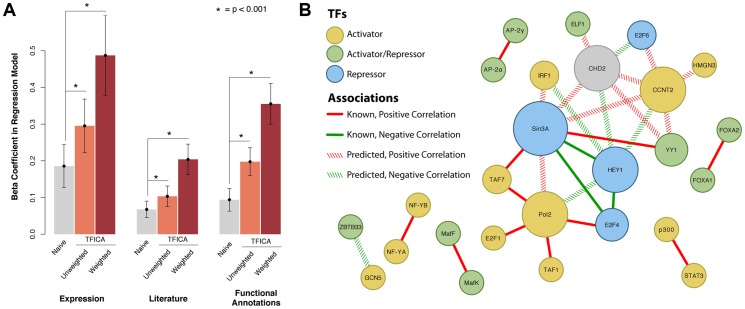
Predicting TF-TF interactions using shared modules as a measure of shared function. (A): Prediction of (i) gene expression correlation, (ii) literature mentions, and (iii) shared functional annotations using a Naive approach, shared TFICA modules, and weighted TFICA modules. The Naive approach (“Naive”) links TFs to TFs by the similarity of their ChIP-Seq targets, “TFICA” links TFs to TFs by the similarity of their significantly associated modules, and weighted TFICA weights these modules in the similarity by their confidence. β coefficients in a linear model are shown with 95% confidence intervals. In each case, TFICA and weighted TFICA significantly outperforms the Naive approach. In addition, we used permutation testing to validate these results. In each case (expression, literature, function) the β coefficient for the permuted model was not significant (β_exp_ = 0.16; 95%CI −0.02–0.34; β_lit_ = −0.02 95%CI −0.08–0.05; β_fun_ = −0.04 95%CI −0.14–0.06, P>0.05 for each). Data not drawn. (B): The top 30 highest-scoring pairs are shown, as measured by target module similarity, 14 of which are known associations (solid lines). Many of these factors form a tight sub-network of activators and repressors.

As we have demonstrated, we can significantly improve the identification of TF targets using TFICA modules. Therefore, we hypothesized that TFs that target overlapping modules may function together to regulate gene expression. We found 3,696 transcription factor pairs (comprising 135 individual TFs) that share a significant proportion of target modules (adjusted p<0.01, Fisher's exact test; Table S3). We assessed the putative TF-TF interactions predicted by TFICA using expression correlation, literature co-occurrences, and shared functional annotation. We compared the predictions of two TFICA similarity metrics (simple Tanimoto and a weighted approach which places more emphasis on higher confidence TF-module pairs) to those from a naive method of simply intersecting ChIP-Seq targets. We evaluated using multivariate linear models and assessed significance with an ANOVA (Figure 3A). Both TFICA approaches outperform the naive method in all 3 evaluations (Figure 3A) with weighted TFICA exhibiting the best performance. In addition, the combined model of both TFICA-similarity plus shared targets significantly outperforms the naive approach alone in all three of these metrics (Figure 3A and Figure S6). TF pairs from TFICA are significantly (1) more correlated in their overall gene expression across the compendium (simple: F = 28.6, p = 1.03e-7; weighted: F = 41.2, p = 1.75e-10), (2) more likely to co-occur in Pubmed abstracts (simple: F = 22.0, p = 2.92e-6; weighted: F = 57.2, p = 6.51e-14), and (3) more likely to share functional annotations (simple: F = 67.0 p = 5.24e-16; weighted: F = 119.4, p = 6.50e-27).

Of the top 30 pairs ranked TF-TF pairs according to module similarity, 14 have been previously reported, such as NF-YA and NF-YB, as well as Pol2 with a number of other initiating factors (Figure 3B). Many of the unreported results may be due to sparse annotation of individual genes (e.g. CHD2, CCNT2, and HEY1), and may indicate new biological links. For example, CHD2 clusters with CCNT2 and Sin3a, which are known cell cycle regulators. CHD2 has previously been proposed as involved in the cell cycle [19] consistent with its role as a DNA damage signaling protein.

### Modules associate transcription factors with disease

**Figure 4:**
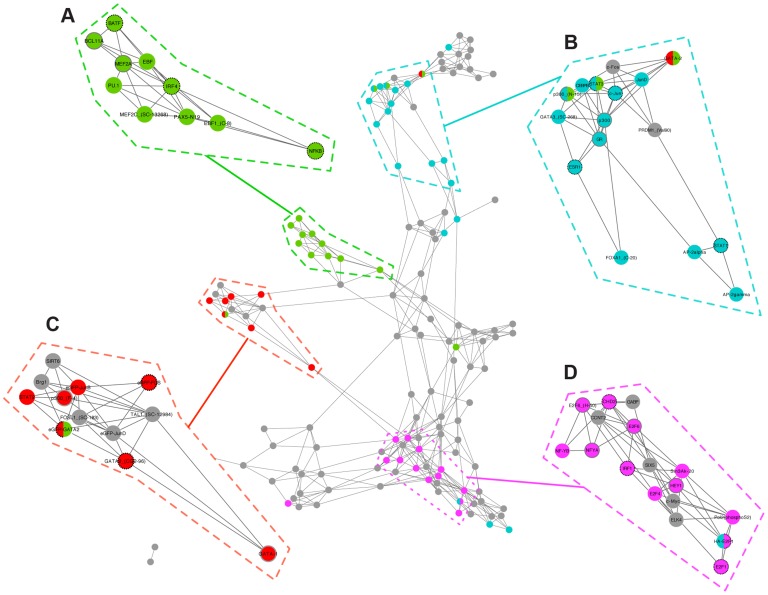
Transcription factor interaction network reveals functional and disease sub-networks. Transcription factors are connected solely on the basis of the similarity of the modules that they regulate. Transcription factors are colored according to a selection of diseases; (A, green): AIDS; (B, blue): arrhythmia; (C, pink): breast cancer; (D, red): hemorrhage. Nodes are annotated with strong (dashed black borders) and weak (solid grey borders) literature support. See Table 2 for details.

We used enrichment analysis to associate ICA functional modules to diseases from the Gene Association Database [20]. Combined with the TFICA analysis, these two datasets allow us to create a transcription factor-disease network. We created a network of 143 transcription factors connected by their targeted functional modules (note that this network is naive to any disease associations). TFs clustered together according to the diseases with which they are significantly associated (Figure 4). In total, we found 7,808 significant associations between 141 transcription factors and 253 diseases. The average number of diseases we associate to a transcription factor is 36 (Figure S8A) with four transcription factors having just one significant disease association (e.g. BAF170 is associated with macular degeneration) and p300 associated with the most (204) diseases. The number of diseases associated with a transcription factor was significantly related to both the number of targets (Figure S8B; Spearman ρ = 0.47, P = 1.2e-7) and the number of GO annotations for the factor (Figure S8C; Spearman ρ = 0.39, P = 1.4e-5). The complete list of significant transcription factor-disease associations is available in Table S4.

Transcription factors with known relationships to disease clustered into distinct groups (Table 2). For example, we found a significant association between module 320 and acquired immunodeficiency syndrome (AIDS) (OR = 12.4; 95% CI = 4.7–28.7; adjusted p = 5.2E-4). Three of the TFs associated with this module (NFKB, IRF4, and BATF) are known to be involved in the transcriptional regulation of human immunodeficiency virus [21]–[23] and cluster together in the interaction network (Figure 4A).

**Table 2:** Transcription factor-disease relationships derived from shared modules.

Disease	Transcription Factor (alt names)	*Significant Components*	TF-Module Odds Ratio	TF-Module P-Value	Disease genes in module/Total disease genes	Relevant Literature Annotations (Pubmed ID)	Literature Support of Association
Arrhythmogenic Right Ventricular Dysplasia	ER-α (ESR1)	*4*	2.71 (2.09, 3.51)	3.1E-12	5/8	Atrial Fibrillation (19860128, 19860128)	strong
						Coronary Heart Disease (20153472)	
						Sudden cardiac death (21658281)	
	GR (NR3C1)	*4*	2.39 (1.84, 3.11)	1.03E-11	5/8	Coronary Heart Disease (19783104)	weak
	P300	*4*	1.75 (1.31, 2.37)	5.9E-05	5/8	Myocardial Infarction (21737953)	weak
	GATA3	*4*	2.02 (1.56, 2.63)	3.89E-08	5/8	Heart Development (18955134)	weak
	c-Jun (JNK)	*4*	1.74 (1.32, 2.31)	2.96E-05	5/8	Myocardial Infarction via K+ channel regulation (20518594)	strong
						Myocardial Infarction (21324895)	
	CEBPB	*4*	1.92 (1.34, 2.83)	0.000141	5/8	Chronic Heart Failure (12601168)	weak
	STAT3	*4*	2.41 (1.79, 3.27)	2.96E-10	5/8	Ventricular Arrhythmias and Contractile Dysfunction (22082679)	strong
						Atrial Fibrillation (18774104)	
	JunD	*4*	1.74 (1.29, 2.37)	0.00011	5/8	Cardiac hypertrophy and Heart failure (15655111)	weak
						Chronic Heart Disease (9136081)	
	STAT1	*4*	1.68 (1.29, 2.19)	9.01E-05	5/8	Atrial Fibrilation (18774104)	strong
						Long QT Syndrome (17490620)	
Breast Cancer	E2F6	*2*	2.29 (1.76, 2.98)	1.21E-10	62/608	Regulation of Tumor Suppressor (ARHI) in BC	weak
		*13*	1.67 (1.31, 2.13)	1.54E-05	26/608		
		*46*	1.75 (1.41, 2.18)	1.91E-07	28/608		
	CHD2	*2*	2.23 (1.72, 2.88)	6.7E-10	62/608	Mammary tumor modifier (17557176)	strong
		*13*	1.82 (1.43, 2.32)	8.28E-07	26/608	Gastric and Colorectal Cancer (21447119)	
						Colon Cancer Biomarker (17390049)	
	NFYA	*2*	2.78 (2.14, 3.60)	9.24E-15	62/608	E2F-1 Regulation (12697671)	weak
						Estrogen Regulation (15224348)	
	IRF1	*2*	2.34 (1.8, 3.05)	3.57E-11	62/608	Resistance to endocrine therapy in Breast Cancer Treatment (22295238)	strong
		*46*	2.91 (2.33, 3.65)	8.9E-23	28/608	Therapy resistant breast tumors (20457620)	
						Commonly mutated/rearranged in breast cancers (19697121, 17498560)	
						Differentially expression in breast tissue >(16241857)	
	HEY1	*2*	2.17 (1.63, 2.92)	1.7E-08	62/608	Target of Notch signaling (18469855)	weak
		*13*	2.5 (1.9, 3.33)	1.39E-12	26/608		
		*46*	2.2 (1.73, 2.81)	8.77E-12	28/608		
	E2F1	*2*	3.31 (2.53, 4.31)	8.36E-18	62/608	Tumor cell growth (22205655)	strong
		*13*	2.58 (1.99, 3.33)	1.27E-12	26/608	E2F1-dependent drug efficacy in breast cancer treatment (22185819, 20215421)	
		*154*	3.78 (2.62, 5.43)	1.61E-12	26/608	Breast cancer treatment (21573702, 21479363)	
						Prognostic breast cancer marker (21453498, 20410059)	
Acquired Immunodeficiency Syndrome	BATF	*320*	3.11 (2.24, 4.35)	4.23E-13	8/40	Inhibits T cell function in HIV (20890291)	strong
	NFKB	*320*	2.53 (1.77, 3.67)	3.04E-08	8/40	HIV use of NFKB pathway (11160127)	strong
						NFKB binds HIV TAR-RNA (22352910)	
	BCL11A	*320*	2.72 (1.99, 3.72)	2.02E-10	8/40	Represses HIV-1 gene transcription (15849318)	weak
	MEF2C	*320*	2.19 (1.55, 3.07)	8.2E-06	8/40	Misregulated in HIV-associated dementia (21170291)	weak
	IRF4	*320*	2.63 (1.92, 3.61)	4.01E-10	8/40	Regulates anti-HIV gene (21078663)	strong
						Expression associated in HIV-related lymphomas (11157493)	
Thrombocytopenia	p300 (CREBBP)	*123*	2.29 (1.5, 3.38)	8.9E-05	5/36	Polymorphisms in p300 associated with thrombocytopenia (18684867)	strong
	Fos	*123*	2.02 (1.47, 2.75)	1.03E-05	5/36	PDGF (c-Fos regulator) knockouts induce thrombocytopenia (12670444)	weak
	GATA1	*123*	1.87 (1.38, 2.53)	2.51E-05	5/36	GATA1 knockout mice develop thrombocytopenia (10216081)	strong

We found a significant association between module 4 and arrhythmogenic right ventricular dysplasia (OR = 82.2, 95% CI: 15.5–531.4, adjusted p = 1.1e-5). ER-α, c-Jun, STAT3, and STAT1 are associated with module 4, and all have known relationships with arrhythmias [24]–[28] and clustered together (Figure 4B). Thus, our network supports the previous suggestion that ER-α may be a promising prognostic marker for the development of atrial fibrillation [24]. In addition, we found that module 123 was significantly enriched for genes associated with thrombocytopenia (OR = 9.9; 95% CI = 2.89–27; adjusted p = 0.022). A number of TFs independently associated with thrombocytopenia, including p300 and GATA1,cluster together in the interaction network and are associated with module 123 (Figure 4C).

Finally, for breast cancer, we found significant associations with modules 2, 13, 46, and 154 with odds ratios of 8.6 (95% CI 5.6–13.4, adjusted p = 2.3e-19), 3.1 (1.8–5.2, adjusted p = 0.004), 3.1 (1.8–5.1, adjusted p = 0.002), and 4.2 (2.4–7.3, adjusted p = 2.1e-4), respectively. Based on their connectivity to these modules, the transcription factors E2F6, CHD2, NFYA, IRF1, HEY1, and E2F1 all cluster together in the TF-TF network (Figure 4D).

### Evaluation on independent data sets shows improved transcription factor disease associations

We performed an evaluation of our TF-disease associations by comparing our derived associations to an independent standard created by combining (1) 37 transcription factor-disease associations from the GWAS Catalog, and (2) 46 associations from OMIM (see Methods). We assessed overall performance using the Area Under the Receiver Operating Characteristic Curve (AUROC). TFICA achieved an AUROC of 0.712 on this test dataset (Figure S7). For comparison, we also evaluated two control strategies: (1) a simple enrichment analysis on the ChIP-Seq targets associated with each transcription factor, and (2) the GREAT tool for annotation cis-regulatory elements in the genome [29]. The simple approach achieved an AUROC of 0.612, whereas GREAT achieved 0.687 (Figure S7). When combined together in a logistic regression model, TFICA significantly improved the performance of GREAT, increasing the AUROC from 0.687 to 0.761 (+10.7%, Chi-Squared = 19, P = 1.1E-5), suggesting the two approaches are complementary. Finally, we repeated this analysis using the AUROC50 which is a common measure used to evaluate performance at low false positive rates (FPR<0.5). We found an AUROC50 value of 0.185 for the naive metric, 0.248 for GREAT, 0.253 for TFICA, and 0.292 for the combined metric, indicating again that TFICA is adding an independent source of information for TF binding.

### A regulatory network of human disease

**Figure 5:**
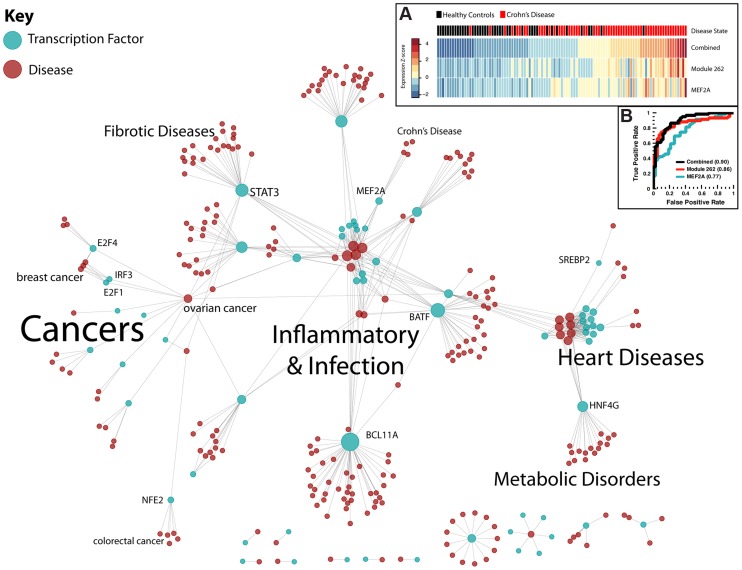
Regulatory network of human disease. Transcription factors (blue) are connected to diseases (red) through modules in this bipartite graph. Prominent clusters of diseases are highlighted, as well as some highly-connected transcription factors. Importantly, STAT3 is connected to many fibrotic diseases, while E2F1 and E2F4 are connected to breast and ovarian cancer. (A): Expression of MEF2A and the projection of module 262 are significantly predictive of disease state. Individuals are ranked by their combined score (sum of normalized expression and module projection). (B): ROC curve for prediction of Crohn's disease from MEF2A expression, module 262 projection, and combined metric.

Using the TFICA disease annotations, we visualized the highest confidence transcription factor-disease associations (see *Materials and Methods*) in a regulatory network connecting 62 transcription factors to 253 human diseases (Figure 5). As expected, substantial parts of this network reflect known biology. For example, TFICA associates HNF4G with metabolic disorders, which corresponds to its KEGG annotation. STAT3's role in fibrotic diseases is well-studied, as it is implicated in the proliferation of fibroblasts and excess ECM proteins [30]. In total, the network visualization describes 491 relationships, 33 are known associations according GAD, OMIM, and GWAS Catalog and thus the remaining 458 are potentially novel transcription factor-disease relationships. Transcription factors are connected with an average of 7.9 diseases and each disease was associated with an average of 1.9 transcription factors. The high confidence associations visualized in Figure 5 and all of the significant ICA-derived associations are available in Table S4.

### Expression of MEF2A and its target genes differentiates patients with Crohn's disease from healthy controls

Using our network analysis, we identified MEF2A as the factor with the highest association with Crohn's disease. MEF2A has been previously associated with cardiovascular disease [31], but is not recognized to have a role in inflammatory bowel disease. MEF2A was associated with Crohn's disease through three modules: 69, 262, and 320. We validated this association using an independent expression dataset (not used in the training set) of 59 patients with Crohn's disease and 42 controls [32]. For each of these modules, the genes comprising the module showed significantly higher levels of differential expression between the two groups compared to genes not in one of the modules (P = 0.017, 1.4e-06, and 0.0084, for modules 69, 262, and 320, respectively). We used permutation testing to correct for a potential bias towards higher differential expression for genes contained within functional modules, after which module 262 remained significant (P = 0.025), which includes genes such as STAT4, CCR5, and SMAD3. We found that expression of MEF2A itself was significantly higher in Crohn's disease patients (Fig. 4A, P = 0.0013, Wilcoxon rank-sum test). Additionally, among genes targeted by MEF2A, genes in module 262 also exhibited a higher level of differential expression among patients with Crohn's disease (P = 0.0019).

To investigate the role of module 262 and MEF2A in classification of Crohn's disease, we projected the expression values in the Crohn's dataset to generate an expression value of the module (see *Materials and Methods*) and found an overall higher expression of the module in patients with Crohn's disease (Fig. 4A, P = 4.6e-09, Wilcoxon rank-sum test). We evaluated MEF2A and the module expression for their performance in a disease classifier using area under the receiver operating characteristic curve (AUROC). Both MEF2A and the aggregated expression of module 262 were significantly predictive (P = 0.0012 and P = 4.5e-06, logistic regression) with AUROCs of 0.77 and 0.86, respectively (Fig. 4B). Finally, we combined MEF2A expression and aggregated module expression into a single model and found that this combined statistic outperformed the other two classifiers (F-test p = 5.8e-11, AUROC = 0.90, Fig. 4B).

## Discussion

We present a computationally efficient and conceptually simple method that is useful in linking transcription factors to their targets and to disease as well as derive several novel such relationships. Using current approaches, such an analysis is challenging as TFs in the ENCODE dataset bind near an average of 6,050 genes. Simple enrichment analysis on the full target set often does not reveal coherent functional groups. Factors may exhibit multifaceted functional roles and target genes in very different cellular contexts, and when all of a TF's targets are grouped together, it becomes difficult to isolate these individual contexts. Our method overlays data-driven functional module information – from a large compendium of human gene expression data – on top of TF binding data from ChIP-Seq. We demonstrate that our method (1) significantly improves TF target identification, (2) accurately identifies the functional roles of factors both independently and in combination with another factor, and (3) discovers new disease associations through these functional modules.

We show that TFICA identifies targets that are significantly more functionally coherent than targets identified by naive (peak-based) methods. Importantly, TFICA can identify these targets even in cases that lack strong support from ChIP-Seq binding data (i.e. sites that are not among the strongest bound peaks). We hypothesized that TFICA would be better able to identify targets that, despite lower binding levels, are biologically important. Our “matched” analyses tests this hypothesis and we observe that TF-target functional annotation sharing (Figure 2B) and expression correlation (Figure S5) is higher for TFICA targets than naively identified targets. In fact, despite that stronger binding more tightly couples TF and target expression, the expression correlation among modules identified by TFICA are consistent with those of the “best” module (genes with highest peak scores) until the modules are associated with more than 40 factors. Additionally, by linking TFs to established modules of gene expression we identify genes where binding of the factor is not observed, but instead, the TF is exerting indirect genetic control. In these cases, we hypothesize that the TF may be controlling expression of a module through its direct targets, some of which may be in the module.

Our factor interaction analysis is useful for suggesting the functional roles of TF through guilt by association, particularly for poorly described factors. For example, CHD2 is a helicase whose function remains to be fully understood. In our TF-TF network, we found that CHD2 is connected with the cyclin CCNT2 (Figure 3B), supporting the hypothesis that CHD2 plays a role in cell cycle [19]. It is important to acknowledge that our method for identifying TF-TF interactions, which uses all of the shared modules between two transcription factors may miss those that are unique to particular biological contexts. Future work will be required to model this type of interaction. In spite of this limitation, we identify many known relationships and outperform traditional approaches (Figure 3A).

Further, we were able to recapitulate many known TF-disease associations without any prior knowledge linking the factor to the disease. For example, our factor-disease network (Figure 5) links ER-α to arrhythmogenic right ventricular dysplasia, supporting recent findings that this protein may be used as a prognostic marker [24]. STAT1 and STAT3, which we also associate with arrhythmogenic right ventricular dysplasia, were recently found to be elevated in mice with sustained atrial fibrillation [27]. In fact, we find too many known associations in this network to enumerate here (refer to Table 2 for an annotated sampling of these associations, and Table S4 for the complete list). Furthermore, we found that the number of diseases associated with a given transcription factor varied widely from just one to hundreds. We hypothesize that this is related to the roles that a particular factor may play in different cellular contexts, with more general factors (e.g. p300, GR, and Pol2) associated with more diseases than more specific factors. We tested this by examining the relationship between the number of diseases associated with a TF and two measures of functional diversity: (1) the number of targets found for that TF by ChIP-Seq and (2) the number of unique GO annotations. In both cases, we found significant positive relationships (Figure S8B–C). In addition, our factor-disease network suggests a novel role for MEF2A in Crohn's disease – an association that would not have been found using the naive method (naive adj. p = 1) and that we validated using an independent data set and analysis (Figure 5A–B). Our module analysis suggests MEF2A is promoting inflammatory response via module 262, which includes STAT4, CCR5, and SMAD3.

It is important to note, however, that this approach is dependent on the quality of the functional networks used. Other methods for generating cohesive functional networks, including data-driven approaches (e.g. PPI networks) and knowledge-based (e.g. functional annotations from ontologies), may complement the approach and improve performance. Further, we derived our modules using a set of 9,395 gene expression experiments without regard to the particular context in which the experiment was performed. Focusing this analysis using a contextually specific set of experiments (e.g. only data focused on cardiovascular disease) could provide further specificity to the disease associations that are derived.

Our work dissects transcription factor function by examining associations with specific gene modules derived from a large compendium of human expression experiments under a wide variety of conditions. This approach is generally applicable in cases where the biological function can neither be described by a single gene nor the entire genome, but instead operates at an intermediate level – groups of genes or groups of functional pathways. We demonstrate improved identification of TF targets and construct a regulatory network of human disease. Finally, we find and validate a novel transcription factor-disease association. We make three databases publicly available to the community: (1) a database of 5,002 transcription factor-module associations, (2) a database of 3,696 putative transcription factor interacting pairs, and (3) a database of 7,808 transcription factor-disease relationships. These resources should further enable researchers to explore TF interactions as well as their roles in human disease.

## Materials and Methods

### Data sources

We obtained transcription factor binding data from UCSC, which included 2,750,490 reproducible binding sites from the ENCODE project [7], [8] and 41,972 gene annotations from RefGene (build hg19). 423 gene expression modules (a.k.a. “components”) determined previously from independent component analysis (ICA) are available at https://simtk.org/home/fcanalysis [14]. These modules were derived from a compendium of human gene expression data downloaded from the Gene Expression Omnibus (GEO). All 9,395 Affymetrix Human Genome U133 Plus 2.0 array deposited in GEO as of May 28, 2008 were downloaded. The ICA analysis was performed and published previously [14]. We obtained gene-disease associations from the Gene Association Database [20] and filtered for positive genome-wide and curated associations, as well as diseases with greater than five genes. We then used the NCBO Annotator [33] service to map the disease terms to terms in the Disease Ontology, resulting in 34,392 distinct associations. 4,267 and 5,279 validation associations were downloaded from the NHGRI GWAS catalog [34] (accessed on 3/31/12) and OMIM (http://omim.org), respectively. For both of these datasets, we mapped local disease terms to standardized terms in the Disease Ontology using the annotator, resulting in 1,842 and 9,866 annotations (of which 35 and 46 map to the transcription factors in our dataset), respectively.

### Generating the transcription factor-ICA module network

We mapped transcription factor binding sites to the nearest gene within 100 kb (gene boundaries 100 kb upstream or downstream of the boundaries of the binding site) in the RefGene annotation to determine putative binding targets of each factor (Figure S1). We linked each transcription factor to each module using an enrichment analysis between the target genes of the transcription factor and the genes contained within each module. We used the hypergeometric distribution to model the expected amount of shared genes and a Fisher's Exact test to test the significance of any deviations. Finally, we corrected for multiple hypothesis testing and filtered for transcription factor-module pairs that were significant with an adjusted p-value less than 0.01 and an odds ratio greater than 1. We clustered TFs using complete-linkage clustering and Euclidean distance (Figure S2). In each module, TFs were ranked by number of genes in the module bound, and a greedy approach was used to determine how many TFs were necessary to account for genes in the module (Figure S3).

We associated each module with between 1 and 121 transcription factors. Our hypothesis is that those that are associated with fewer TFs will be of higher confidence than those associated with many, which may contain more noise. We therefore identified two subsets of TF-module pairs: (i) a “high-confidence” set of TF-module pairs where the module was only associated with one TF and (ii) a set of “medium-confidence” TF-module pairs where the module was associated to at most three TFs. We use these subsets in our evaluation of the TFICA TF-target predictions we make through the associated modules. Additionally, we computed the (Spearman) expression correlations between all TFs and all modules (projections) and used this distribution of correlations as an empirical null distribution. The correlations from the 5,002 significantly overlapping TF-module pairs were z-transformed to this distribution to determine the number of pairs that were empirically significantly correlated at the expression level.

For each of the 9,395 gene expression datasets, we projected the (log-transformed) gene expression measurements of each gene onto the loadings for each module. In cases where the module contained the transcription factor itself, we removed the transcription factor prior to computing the prediction. For modules with significant associations with at most three transcription factors, we tested the (Spearman) correlation between the projection and the logged gene expression value of the transcription factor.

We evaluated our TFICA approach by comparing to two different naive approaches: a “best” naive approach consisting of a set of equal size to the module and comprised of the top bound genes (i.e. those with the highest ChIP-Seq scores), and a “matched” naive approach, which is an equal-sized set with randomly chosen targets matched to the score distribution of the targets in the component. We then assessed whether TF expression is better correlated with projections from modules from TFICA (from above) compared to the average expression of the genes in these naive modules. We segregated by number of transcription factors associated with each module and presented how often TFICA outperforms the naive method for that “number of TFs” threshold. Additionally, we performed a paired t-test at each threshold comparing the r^2^ values between TF-module correlations from TFICA and the naive methods. Finally, we examined the proportion of TF-target pairs that share functional annotations based on the hypothesis that true regulatory targets are more likely to share these annotations. We split the TFICA associations into three groups: (i) high confidence associations, (ii) medium confidence associations, and (iii) all associations. We compared each of these three groups to the naive approach.

### Building the transcription factor interaction network

We used the Tanimoto coefficient as a measure of similarity between each pair of transcription factors. The Tanimoto coefficient is a common set similarity comparison technique used to compare two sets. It is defined as the size of the intersection of the two sets divided by the size of the union of the two sets. In this case, the sets were the modules that were significantly associated with each transcription factor. We used these similarities to build two transcription factor interaction networks. The first was a small network of the top 30 interactions based on the tanimoto coefficient (Figure 3B). We annotated this network with curated information on the function (activator or repressor) and biological interactions between these factors. This network also visualizes the pairwise Pearson's correlation between the expression pattern across the 9,395 gene expression experiments in the compendium.

The second network contains 140 factors and was generated by connecting transcription factors whose pairwise tanimoto score was greater than 0.2. We used Cytoscape [35] (version 2.8.2) and the force-directed weighted layout visualize this network. To test the significance between the number of shared modules between each pair of transcription factors, we used enrichment analysis using the hypergeometric distribution and correction (adjusted p<0.01 and odds ratio >1).

### Evaluation of TFICA TF-TF network versus other approaches

We evaluated our TFICA TF-TF network against a naive approach which linked TFs simply by their shared ChIP-Seq targets. In the naive approach, we used Tanimoto similarity to quantify the relationship between each pair of TFs. For TFICA TF similarity we used two approaches: (i) the Tanimoto similarity between the TFICA modules associated with each TF and (ii) a weighted version of the Tanimoto similarity between the TFICA modules associated with each TF. In the weighted version we upweighted high confidence TF-module associations over low-confidence. We believe that the fewer TFs a given module is associated with the higher the confidence. We used the following equation to generate a weighted Tanimoto similarity score for each TF-TF pair:
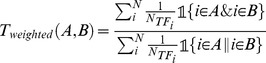
Where *A* and *B* are the sets of modules associated with the two factors, *N* is the total number of modules, and *N_TFi_* is the total number of TFs significantly associated with module *i*. We compared these three TF-TF similarity values for their relationship with correlation in TF expression, co-reporting in literature, and shared functional annotations. For each, we fit a linear model and report the β coefficients, confidence intervals, and p-values. We directly compared the TFICA approaches to the naive approach using an ANOVA.

### Construction of the transcription factor-disease network

We associated the derived 423 ICA modules to the 632 diseases reported in GAD (Genetic Association Database) by analyzing the overlap of the genes associated with a given disease with the genes in a given module. We used enrichment analysis with correction (FDR) for multiplicity to determine significant overlaps. Previously we linked factors to modules to produce TF-module pairs. From this analysis, we have module-disease pairs and thus, we can link TFs to diseases through their shared modules. For example, if a TF significantly targets a module and that module has a significant proportion of a disease's known genes, then we predict a relationship between the TF and the disease.

We generated two lists of transcription factor disease associations. The first contains all significant associations (adjusted p<0.01 and odds ratio >1), which was used for validation (below). The second is a small set of high confidence associations, which included a stringent set of associations (odds ratio >3, adjusted p<0.001), as well as the best association with adjusted p<0.01 and odds ratio greater than 1 for those diseases that had no “stringent” associations used for visualization. We used Cytoscape [35] (version 2.8.2) and the spring embedded layout to visualize the resulting network.

In order to assess the predictive performance of the algorithm we needed to optimize the alpha level for determining significance between modules and diseases. We used a subset of 632 associations from the GAD that are for transcriptions factors directly to calibrate this parameter. We computed the AUROC at a range of thresholds (Figure S7A) to determine the optimal value (determined to be adjusted FDR = 0.15).

We tested the performance of our method (using module-disease p value) against an independent validation dataset of known disease to gene (and thus, disease to TF) associations, a combination of the NHGRI GWAS catalog and OMIM (Figure S7B). We compared the performance to a naive method, where we evaluated the enrichment between the raw transcription factor targets and known disease genes (Fisher's test p value was used as a predictor). Additionally, we compared TFICA to an established method for determining enrichment for particular genomic annotations, GREAT (binomial p value was used as a predictor) [29]. We used the AUROC and AUROC50 values as summary statistics of the predictive performance of the methods. We combined the predictors from our method and GREAT using logistic regression and used an ANOVA to assess the additive contribution of our approach to GREAT testing significance with a Chi-Squared test. Comparisons of ROC curves were performed using DeLong's method in the pROC package for R.

### Validation of association between MEF2A and Crohn's disease

We obtained a publicly-available expression dataset for 59 Crohn's patients and 42 healthy controls (GEO Accession Number: GSE3365) [32]. Gene expression measurements were determined using the median measurement of all probes in the gene. For each gene, differential expression between cases and controls was determined using a Student's t-test. The distributions of absolute values of these t-statistics were tested using a Student's t-test/Wilcoxon rank-sum test to determine significant differences in differential expression of gene sets.

To generate an expression value for the module, a projection was calculated, using the dot product of the expression values of each gene in the module and their individual loadings derived from ICA. Expression of MEF2A and the projection of the module were normalized to a z-score and summed to create a “combined” metric. These three metrics were compared among cases and controls using a Wilcoxon rank-sum test and fit using a logistic regression (binomial).

### Statistical analysis

All statistical analyses were performed with the R statistical computing package (version 2.14.1). Enrichment analyses were computed using Fisher's exact test, with p-values corrected using the Benjamini-Hochberg FDR correction for multiple hypotheses. All expression correlations were performed using Spearman correlations. ROC curves were generated using the ROCR package for R [36] and comparisons of ROC curves were performed using DeLong's method in the pROC package for R.

## Supporting Information

Figure S1 **Method details.** (A): The distance between TF binding sites from ENCODE and the nearest gene in RefGene are shown. (B): The distribution of genes putatively regulated by each of the transcription factors (TFs) on the basis of their proximity is shown. Many transcription factors map to thousands or tens of thousands of genes. (C): Enrichment between genes regulated by each TF and genes found in modules generated by ICA results in 5,002 TF-module associations. These modules are associated with up to 121 TFs.
(PDF)

Figure S2 **TF-module associations.** Enrichments between transcription factor target sets and genes found in each module from ICA are plotted and hierarchically clustered by TF and module. The most striking cluster along modules is module 57 (red box), which includes many transcription factors as targets themselves. See Table 1 for further description of modules and Table S1 for the full dataset. (A): The association between ZNF274, SETDB1, and KAP1 is shown in module 111, which includes many zinc finger genes. (B): Module 158 contains many fatty acid synthesis genes and is significantly enriched for targets of SREBP1 and SREBP2. (C): The complex association between GCN5, GTF2B, NELFe, and others with modules 104 and 62 is shown.
(PDF)

Figure S3 **TFs can explain modules identified by ICA.** The number of significantly enriched transcription factors that are required to regulate (A) 80%, (B) 90%, and (C) 100% of all possible genes in a module. Possible genes are defined as genes that are targeted – as determined by ChIP-Seq experimental data – by at least one of the 148 TFs in the dataset. 87 modules could not be explained by targets of any associated TF (shown in the N/A column). Only significant TF-module associations are used to calculate TFs required. (D) A histogram of the proportion of the modules that are “explainable” by TF targets determined by ChIP-Seq.
(PDF)

Figure S4 **Variance explained by modules.** The number of transcription factors significantly associated with each module correlates with (A) the rank of the module when sorted by the module's variance (r = −0.296, p = 4.8e-10) and (B) the percent of total variance of that module (r = 0.265, p = 2.8e-08).
(PDF)

Figure S5 **TFICA outperforms naive modules in expression correlation.** For each of the 9,395 gene expression experiments, the expression values of each gene in a module are projected using the ICA loadings. For each TF-module pair tested, this projection is compared to the mean expression of two modules based directly on ChIP-Seq data: a “best” naive module, a set of genes (the same number as the TFICA module) with the highest ChIP-Seq binding scores (black), and another equally-sized “matched” set with binding scores matched to the scores of bound genes in the module (red). These comparisons are separated on the basis of modules associated with a varying number of transcription factors. (A): The proportion of cases where TFICA is more correlated than expression than each of the two naive modules. Diamonds indicate significantly higher differences, as determined by a binomial test. Note that TFICA outperforms the “matched” module at every threshold, and the “best” module at high- and medium-confidence associations (one and three or fewer TFs per module; see text). (B–C): The correlation values at each threshold are compared using a t-test and the one-sided p-value where the TFICA correlation is higher (B) and lower (C) is shown here. (D): The number of significant TF-module pairs in each bin are plotted.
(PDF)

Figure S6 **TF-TF interaction prediction performance comparison.** Similarity of target modules among TF pairs is correlated with gene expression correlation (top row), literature co-reporting (middle row), and shared functional annotations (bottom row): We compared three approaches: (i) a naive similarity approach based on the proportion of targets two TFs share (left column), (ii) a TFICA approach based on the proportion of significant modules two TFs share (middle column), and (iii) a TFICA approach where the TF-module were weighted by the confidence of the association (right column). In each case the weighted method is most correlated, followed by the non-weighted TFICA method, with the naive approach being the least correlated.
(PDF)

Figure S7 **Performance assessment.** (A): A number of module-disease FDR cutoffs were assessed against a training dataset of associations from GAD to train our method (B): The TFICA method (red) identifies TF-disease associations, which are compared to enrichments using GREAT (blue), as well as a simple target enrichment method (black). Performance is visualized using ROC curves using a combination of the NHGRI GWAS catalog and OMIM as a gold standard dataset. A composite measuring using our method and GREAT is shown in purple.
(PDF)

Figure S8 **Associated diseases by TF.** A histogram of the number of TFs associated with a given number of diseases is shown in (a). Additionally, Two estimates of global function are plotted: (b) the number of GO annotations for a given TF and (c) the number of ChIP-Seq targets for a given TF. In both cases the number of diseases associated to a TF using the TFICA algorithm is significantly correlated to the TF's diversity of function.
(PDF)

Table S1 **Significant associations between transcription factors and modules.**
(CSV)

Table S2 **Projections of gene expression measurements for module-TF pairs.**
(CSV)

Table S3 **Modules shared, expression correlation, and literature evidence between pairs of transcription factors.**
(CSV)

Table S4 **A regulatory network of human disease.**
(CSV)
