# Supplementary figures and images for: Coherent Functional Modules Improve Transcription Factor Target Identification, Cooperativity Prediction, and Disease Association
Source: PLoS Genet. 2014 Feb 6;10(2):e1004122. doi: 10.1371/journal.pgen.1004122 (PMC3916285; doi:10.1371/journal.pgen.1004122)

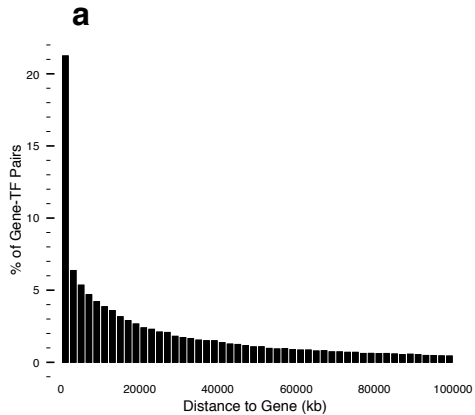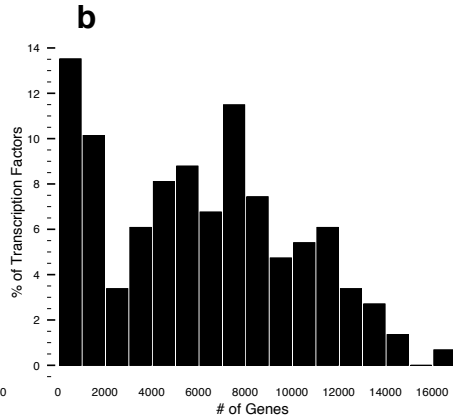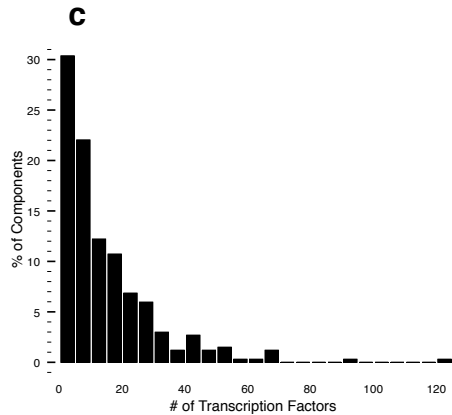

Supplement: Figure S1 — Method details. (A): The distance between TF binding sites from ENCODE and the nearest gene in RefGene are shown. (B): The distribution of genes putatively regulated by each of the transcription factors (TFs) on the basis of their proximity is shown. Many transcription factors map to thousands or tens of thousands of genes. (C): Enrichment between genes regulated by each TF and genes found in modules generated by ICA results in 5,002 TF-module associations. These modules are associated with up to 121 TFs. (PDF) [file pgen.1004122.s001.pdf]

# ICA Components

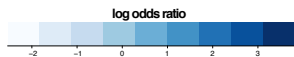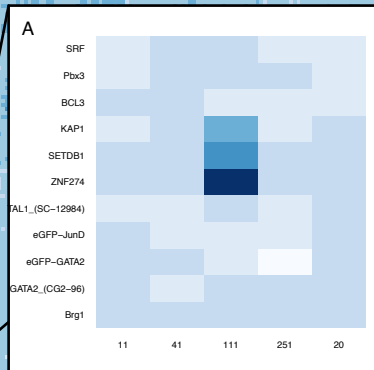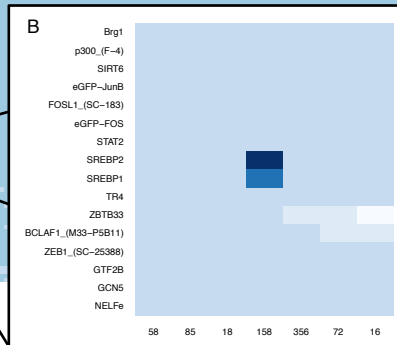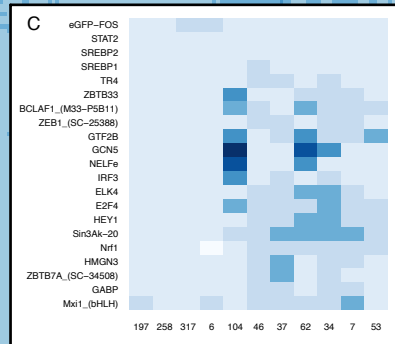

Supplement: Figure S2 — TF-module associations. Enrichments between transcription factor target sets and genes found in each module from ICA are plotted and hierarchically clustered by TF and module. The most striking cluster along modules is module 57 (red box), which includes many transcription factors as targets themselves. See Table 1 for further description of modules and Table S1 for the full dataset. (A): The association between ZNF274, SETDB1, and KAP1 is shown in module 111, which includes many zinc finger genes. (B): Module 158 contains many fatty acid synthesis genes and is significantly enriched for targets of SREBP1 and SREBP2. (C): The complex association between GCN5, GTF2B, NELFe, and others with modules 104 and 62 is shown. (PDF) [file pgen.1004122.s002.pdf]

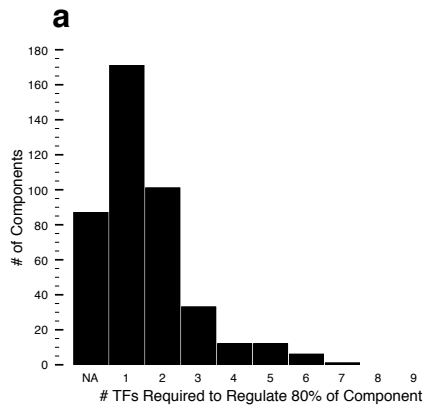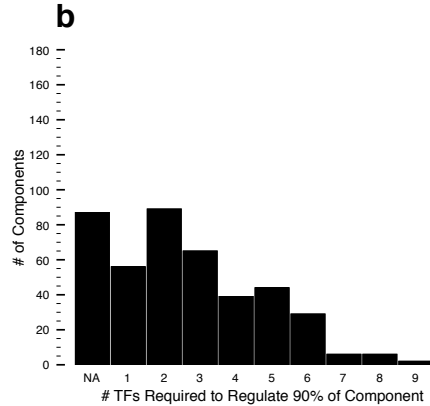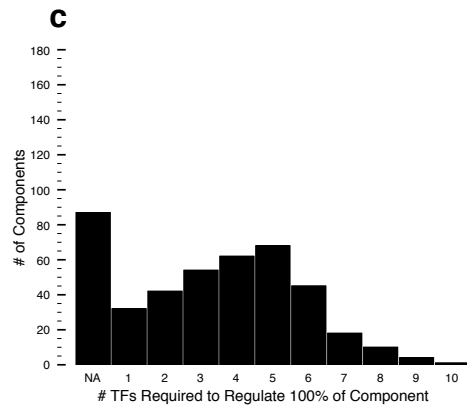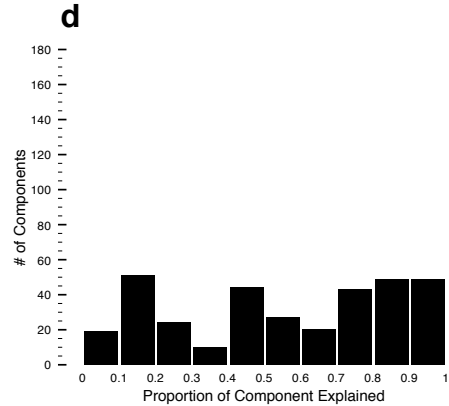

Supplement: Figure S3 — TFs can explain modules identified by ICA. The number of significantly enriched transcription factors that are required to regulate (A) 80%, (B) 90%, and (C) 100% of all possible genes in a module. Possible genes are defined as genes that are targeted – as determined by ChIP-Seq experimental data – by at least one of the 148 TFs in the dataset. 87 modules could not be explained by targets of any associated TF (shown in the N/A column). Only significant TF-module associations are used to calculate TFs required. (D) A histogram of the proportion of the modules that are “explainable” by TF targets determined by ChIP-Seq. (PDF) [file pgen.1004122.s003.pdf]

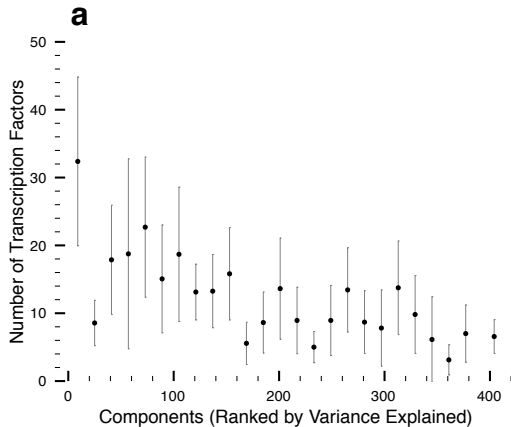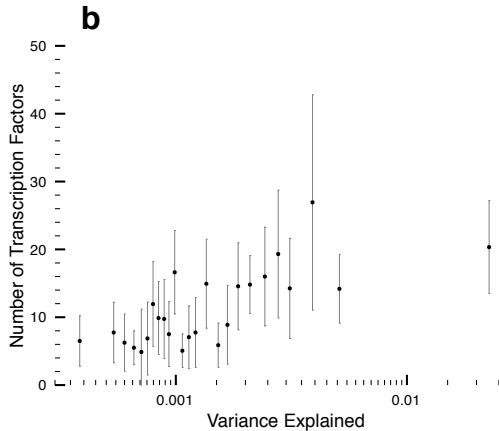

Supplement: Figure S4 — Variance explained by modules. The number of transcription factors significantly associated with each module correlates with (A) the rank of the module when sorted by the module's variance (r = −0.296, p = 4.8e-10) and (B) the percent of total variance of that module (r = 0.265, p = 2.8e-08). (PDF) [file pgen.1004122.s004.pdf]

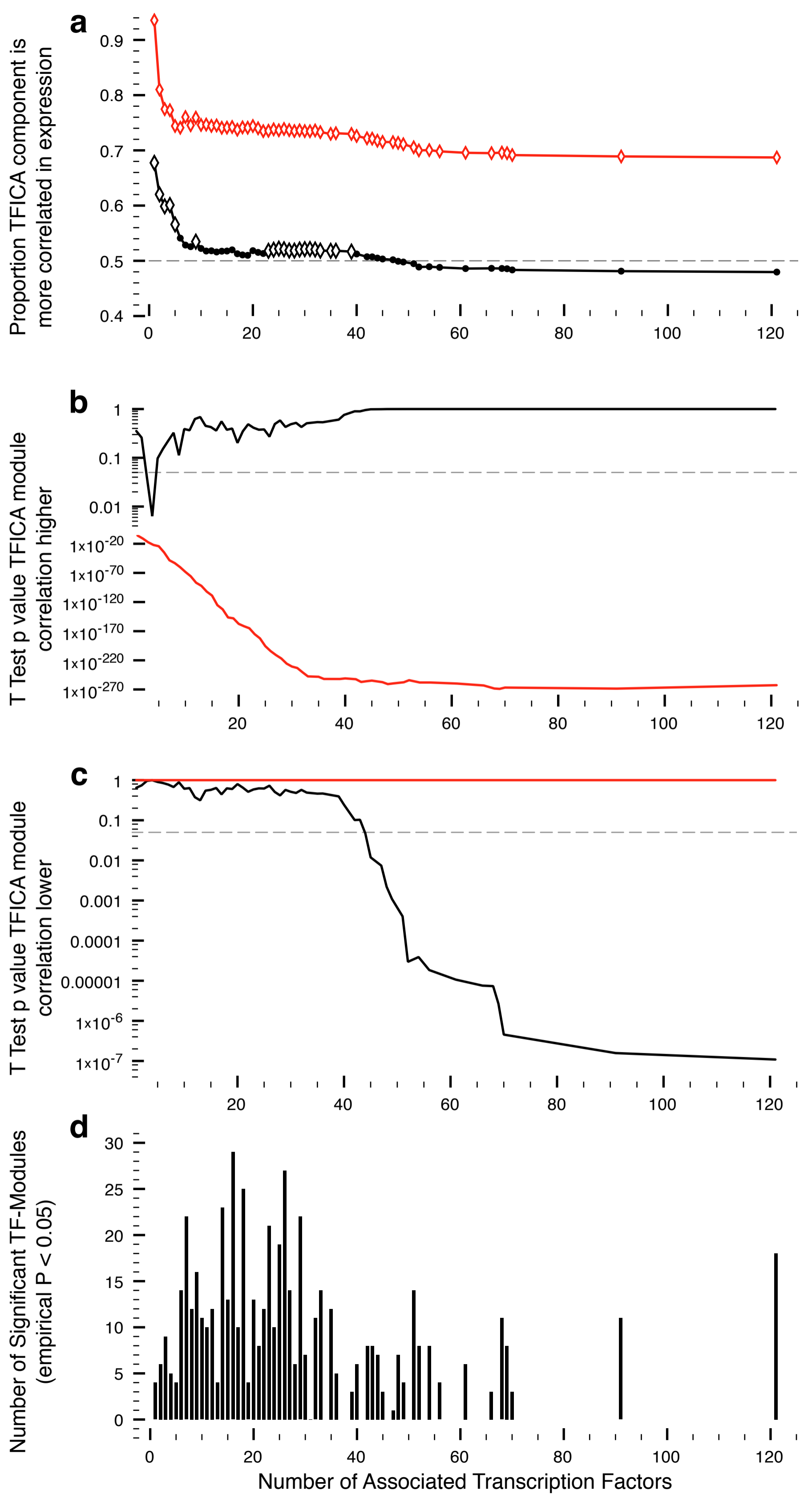

Supplement: Figure S5 — TFICA outperforms naive modules in expression correlation. For each of the 9,395 gene expression experiments, the expression values of each gene in a module are projected using the ICA loadings. For each TF-module pair tested, this projection is compared to the mean expression of two modules based directly on ChIP-Seq data: a “best” naive module, a set of genes (the same number as the TFICA module) with the highest ChIP-Seq binding scores (black), and another equally-sized “matched” set with binding scores matched to the scores of bound genes in the module (red). These comparisons are separated on the basis of modules associated with a varying number of transcription factors. (A): The proportion of cases where TFICA is more correlated than expression than each of the two naive modules. Diamonds indicate significantly higher differences, as determined by a binomial test. Note that TFICA outperforms the “matched” module at every threshold, and the “best” module at high- and medium-confidence associations (one and three or fewer TFs per module; see text). (B–C): The correlation values at each threshold are compared using a t-test and the one-sided p-value where the TFICA correlation is higher (B) and lower (C) is shown here. (D): The number of significant TF-module pairs in each bin are plotted. (PDF) [file pgen.1004122.s005.pdf]

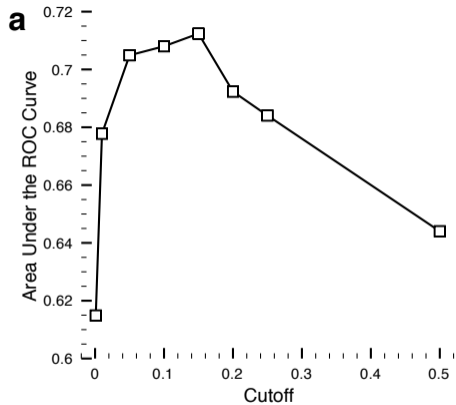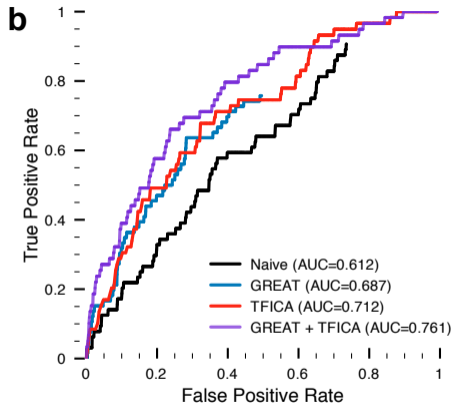

Supplement: Figure S7 — Performance assessment. (A): A number of module-disease FDR cutoffs were assessed against a training dataset of associations from GAD to train our method (B): The TFICA method (red) identifies TF-disease associations, which are compared to enrichments using GREAT (blue), as well as a simple target enrichment method (black). Performance is visualized using ROC curves using a combination of the NHGRI GWAS catalog and OMIM as a gold standard dataset. A composite measuring using our method and GREAT is shown in purple. (PDF) [file pgen.1004122.s007.pdf]

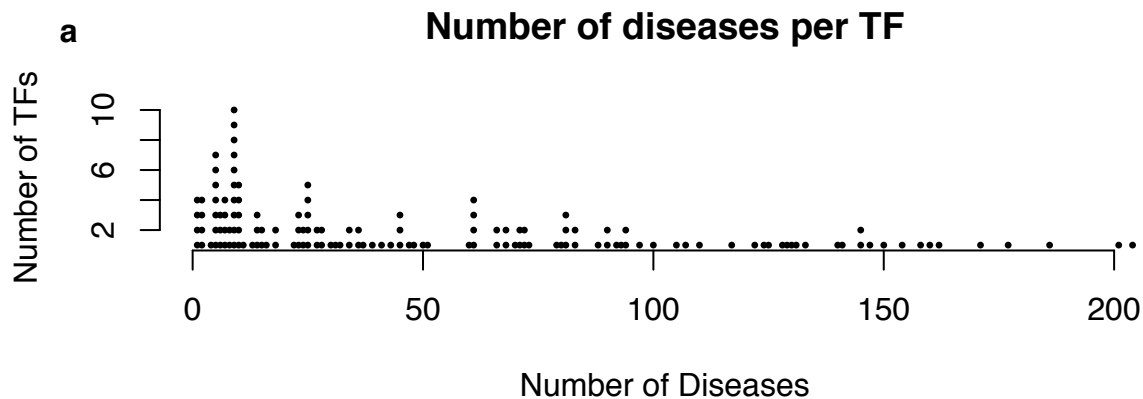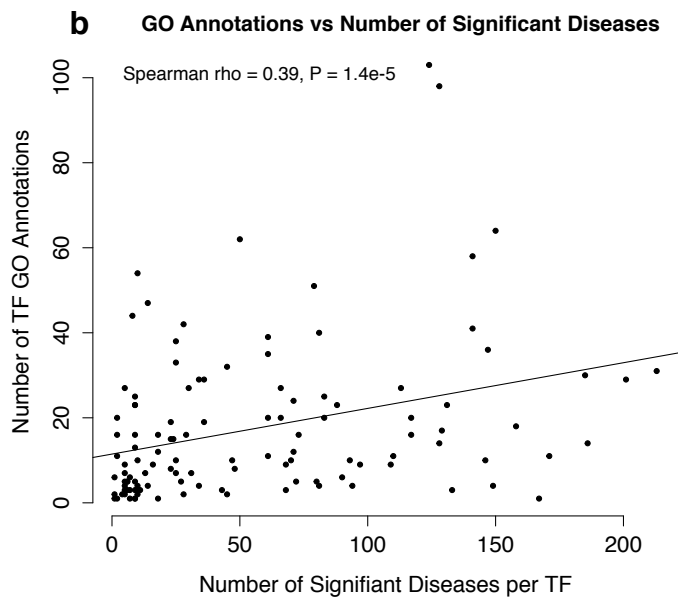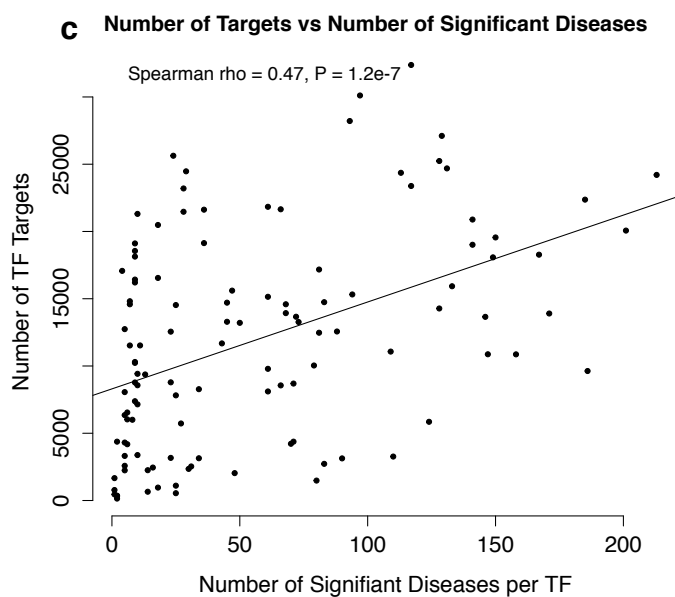

Supplement: Figure S8 — Associated diseases by TF. A histogram of the number of TFs associated with a given number of diseases is shown in (a). Additionally, Two estimates of global function are plotted: (b) the number of GO annotations for a given TF and (c) the number of ChIP-Seq targets for a given TF. In both cases the number of diseases associated to a TF using the TFICA algorithm is significantly correlated to the TF's diversity of function. (PDF) [file pgen.1004122.s008.pdf]
