# Supplementary material for: Coherent Functional Modules Improve Transcription Factor Target Identification, Cooperativity Prediction, and Disease Association
Source: PLoS Genet. 2014 Feb 6;10(2):e1004122. doi: 10.1371/journal.pgen.1004122 (PMC3916285; doi:10.1371/journal.pgen.1004122)
Supplement: Figure S6 — TF-TF interaction prediction performance comparison. Similarity of target modules among TF pairs is correlated with gene expression correlation (top row), literature co-reporting (middle row), and shared functional annotations (bottom row): We compared three approaches: (i) a naive similarity approach based on the proportion of targets two TFs share (left column), (ii) a TFICA approach based on the proportion of significant modules two TFs share (middle column), and (iii) a TFICA approach where the TF-module were weighted by the confidence of the association (right column). In each case the weighted method is most correlated, followed by the non-weighted TFICA method, with the naive approach being the least correlated. (PDF) [file pgen.1004122.s006.pdf]

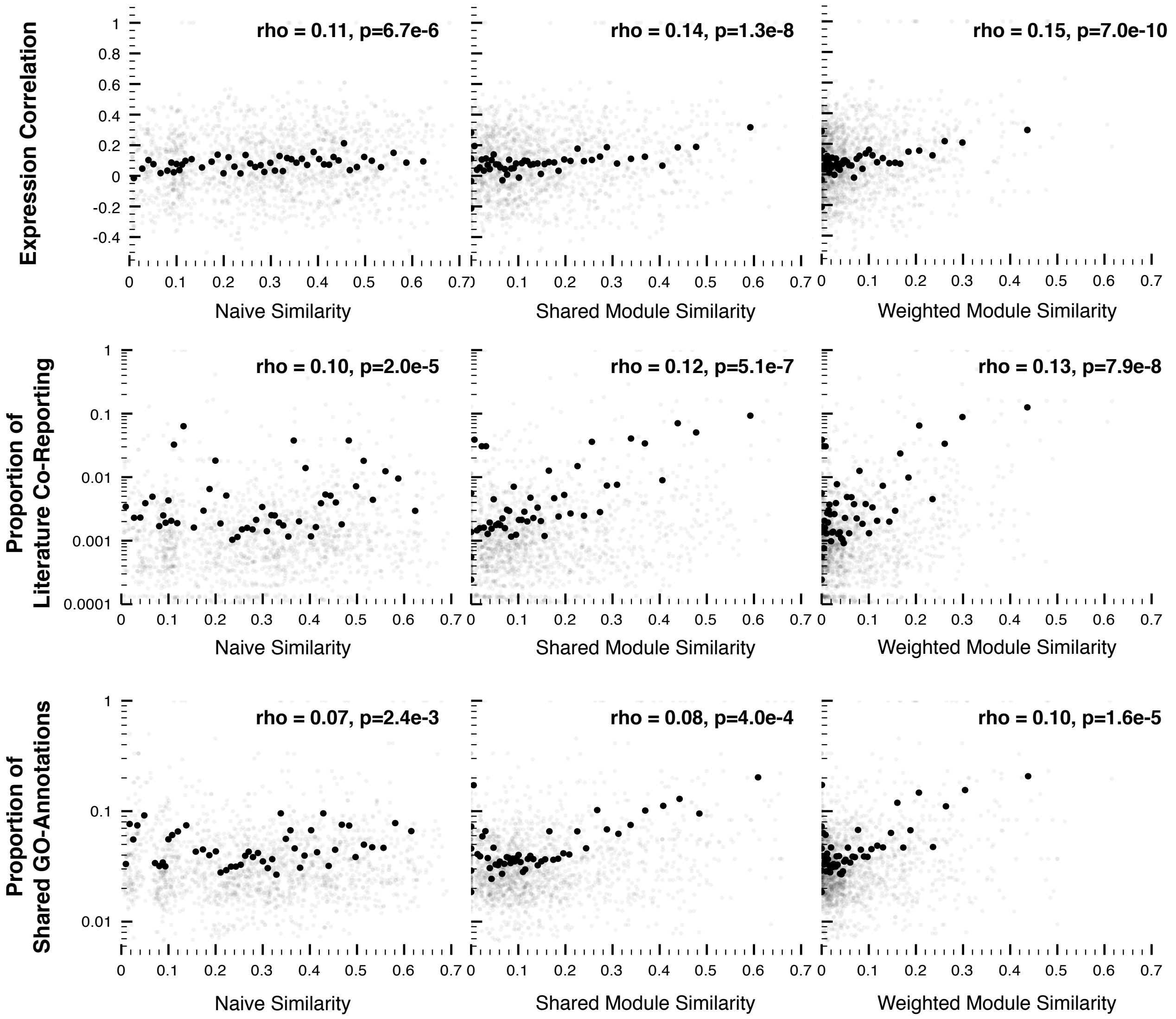

Expression Correlation
rho = 0.11, p=6.7e-6
rho = 0.14, p=1.3e-8
rho = 0.15, p=7.0e-10
Naive Similarity
Shared Module Similarity
Weighted Module Similarity
Proportion of
Literature Co-Reporting
rho = 0.10, p=2.0e-5
rho = 0.12, p=5.1e-7
rho = 0.13, p=7.9e-8
Proportion of
Shared GO-Annotations
rho = 0.07, p=2.4e-3
rho = 0.08, p=4.0e-4
rho = 0.10, p=1.6e-5
